# The Uterine Melatonergic Systems of AANAT and Melatonin Membrane Receptor 2 (MT2) Are Essential for Endometrial Receptivity and Early Implantation in Mice

**DOI:** 10.3390/ijms24087127

**Published:** 2023-04-12

**Authors:** Xiao Ma, Jing Wang, Likai Wang, Laiqing Yan, Yunjie Liu, Wenkui Ma, Pengyun Ji, Lu Zhang, Guoshi Liu

**Affiliations:** National Engineering Laboratory for Animal Breeding, Key Laboratory of Animal Genetics and Breeding of the Ministry of Agricultural, Beijing Key Laboratory for Animal Genetic Improvement, College of Animal Scienceand Technology, China Agricultural University, Beijing 100193, China

**Keywords:** AANAT, MT2, uterus, endometrial receptivity, embryo implantation

## Abstract

In the current study, using *Aanat* and *Mt2* KO mice, we observed that the preservation of the melatonergic system is essential for successful early pregnancy in mice. We identified that aralkylamine N-acetyltransferase (AANAT), melatonin receptor 1A (MT1), and melatonin receptor 1B (MT2) were all expressed in the uterus. Due to the relatively weak expression of MT1 compared to AANAT and MT2, this study focused on AANAT and MT2. *Aanat* and *Mt2* KO significantly reduced the early implantation sites and the abnormal morphology of the endometrium of the uterus. Mechanistical analysis indicated that the melatonergic system is the key player in the induction of the normal nidatory estrogen (E2) response for endometrial receptivity and functions by activating the STAT signaling pathway. Its deficiency impaired the interactions between the endometrium, the placenta, and the embryo. The reduction in melatonin production caused by *Aanat* KO and the impairment of signal transduction caused by *Mt2* KO reduced the uterine MMP-2 and MMP-9 activity, resulting in a hyperproliferative endometrial epithelium. In addition, melatonergic system deficiency also increased the local immunoinflammatory reaction with elevated local proinflammatory cytokines leading to early abortion in the *Mt2* KO mice compared to the WT mice. We believe that the novel data obtained from the mice might apply to other animals including humans. Further investigation into the interaction between the melatonergic system and reproductive effects in different species would be worthwhile.

## 1. Introduction

A critical stage in mammalian reproduction is the implantation of the blastocyst into the uterine cavity [1]. It has been estimated that approximately 30% of human conceptions end up with a successful pregnancy during a given menstrual cycle, and another 50% to 60% of the conceptions only last around 20 weeks of gestation [2,3]. Among the miscarriages, implantation defects contribute to approximately 75% of the cases [4]. Implantation, a prerequisite for successful pregnancy, is a highly coordinated process that requires meticulously timed maternal/embryonic communication [1]. The window of implantation (the time when endometrial receptivity to embryos is at its peak) in humans occurs 7–10 days following ovulation; in mice, it is around 4–4.5 days after coitus (dpc) [1]. The mucosal lining of the uterus, the endometrium, serves as the first interface between a mother and her developing fetus. Endometrial epithelial cells play an important role in maternal–embryonic communication during implantation; stromal cells transform into a secretory cell type (i.e., decidualize) to nourish the growth and development of the early embryo [5]. Inhibition of epithelial cell proliferation and remodeling of stromal cells are two coordinated molecular and histological alterations that occur during the implantation window. These changes are carefully time-dependently coordinated by the steroid hormones estrogen (E2) and progesterone (P4) as well as other growth factors [6,7,8].

The uterus is an extremely active organ. The endometrium goes through a complicated process of mucosal differentiation and immune cell inflow known as decidualization during each cycle to prepare for pregnancy. This process is controlled by shifting hormone cycles. The timely presence of properly functional maternal immune cells supporting proper trophoblast invasion of semi-allogeneic fetal cells and spiral artery remodeling is crucial for the early stages of pregnancy [9,10,11,12]. Both the mother and the fetus may suffer consequences if immunological components are not properly recruited, incited, or activated at the fetal–maternal interface (i.e., leading to preterm labor (PTL), preeclampsia (PE), intrauterine growth restriction (IUGR), and recurrent miscarriages) [10,12,13].

Melatonin (N-acetyl-5-methoxytryptamine, MT) is a pleiotropic molecule. It is produced by the pineal gland and also other tissues and cells to serve as autocrine and paracrine mechanisms [14,15,16]. For example, skin [17,18], lymphocytes [19], and the gastrointestinal system [20] synthesize melatonin locally. In addition, female reproductive organs including the ovaries and the uterus have the capacity to produce melatonin [21]. It is well-documented that melatonin is synthesized in mitochondria [14,22,23]. Therefore, the melatonin level in mitochondria is around 100 times higher than in the plasma. AANAT (aralkylamine N-acetyltransferase) is the rate-limiting enzyme for melatonin synthesis [24]. Two G protein-coupled receptor (GPCR) family members, melatonin receptor 1A (MTNR1A or MT1) and melatonin receptor 1B (MTNR1B or MT2), are responsible for the majority of biological actions of melatonin [25]. Their distributions rely on specific cell types and tissues [26,27,28].

Melatonin has many crucial biological functions including anti-inflammatory, anticancer, anti-obesity, antioxidant activities [15,16]. Here, we will focus on its activities related to female reproduction. For instance, mitochondria of oocytes synthesize melatonin, which enhances oocyte maturation and reduces endoplasmic reticulum stress in pig oocytes [29,30]. It promotes the development of the embryo [15,31,32] in a variety of species including pigs [33], cows [34,35], sheep [36], and mice [37,38,39,40]. Melatonin administration orally increases not only fertilization rates in women using assisted reproductive technologies, but also the survival rate of embryos [41]. Melatonin promotes the luteinization of granulosa cells, the effectiveness of embryo attachment, and the maintenance of pregnancy [16,31,32]. On the other hand, suppressing melatonin production by disrupting the hypothalamic–pituitary–gonadal axis or prolonged photoperiod has a deleterious effect on the sow reproductive performance [16]. Interestingly, melatonin also stimulates the expression of AANAT and MT2 in mouse uteri during early pregnancy, increasing the rate of blastocyst implantation and litter size [42]. Melatonin application stimulates estradiol levels (E2) to enhance implantation in mice [43]. The potential mechanisms are probably that melatonin enhances the uterine microenvironment, boosting the development of antioxidant enzymes including superoxide dismutase (SOD) and catalase (CAT) [44]. Melatonin also upregulates heparin-binding epidermal growth factor-like growth factor (HB-EGF) expression in the endometrium in mice undergoing in vitro fertilization and embryo transfer (IVFET), improving the likelihood of implantation [42,45,46]. MT2 activation impacts its downstream element of P53, which regulates the expression of leukemia inhibitory factor (LIF), thereby improving embryo implantation [46]. In humans, melatonin is transferred from the maternal to the fetal circulation both easily and rapidly. Therefore, using melatonin as an antioxidant is a potential therapeutic agent for patients with preeclampsia [47]. This study was designed as information on the roles of uterus melatonergic systems including the effect of the synthetic rate-limiting enzyme AANAT and its membrane receptors on the endometrial receptivity and implantation is limited. Our preliminary results indicate the direct association between melatonergic systems and endometrial receptivity and implantation. This association is mediated by the elevated levels of endometrium E2/P4 during the window of implantation. We hope that these valuable data will provide another avenue for mammalian reproductive research in the future.

## 2. Results

### 2.1. Identification of Expression of AANAT, MT1, and MT2 in Uteri and Their KO for Mice Fertility

**Figure 1:**
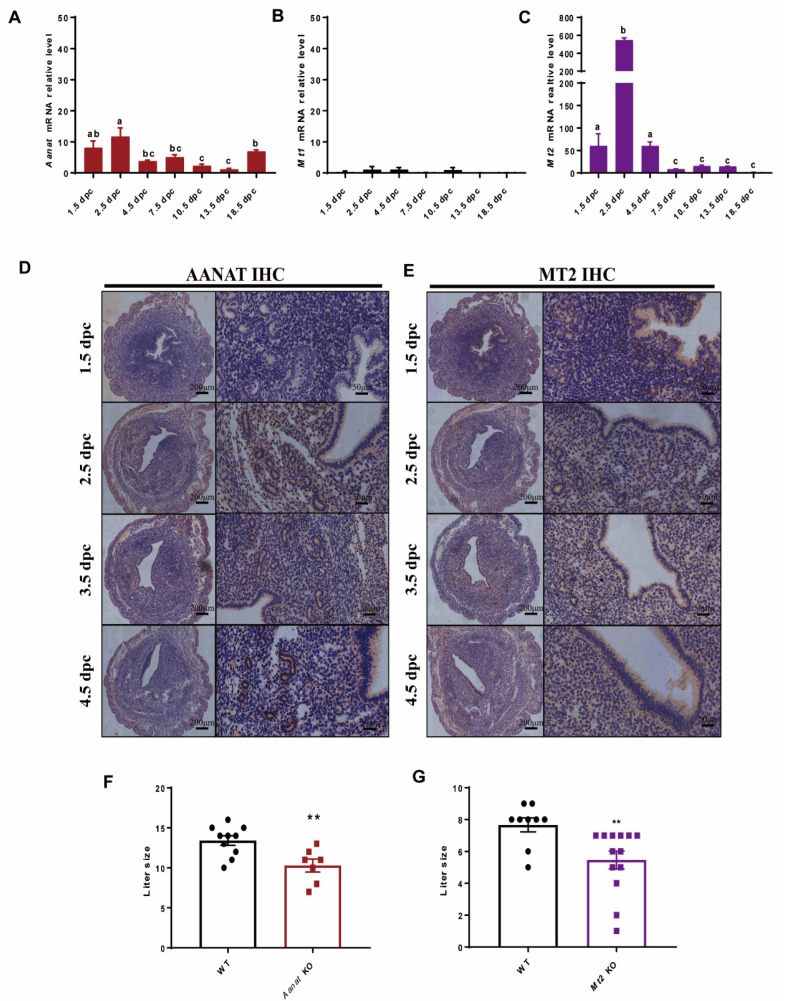
The expression patterns of *Aanat* and *Mt1/2* in the uterus and their KO for mice fertility. (**A**–**C**) *Aanat*, *Mt1*, and *Mt2* expression during pregnancy in mouse uteri. Values with superscript letters a,b,c are significantly different across columns (*p* < 0.05) (**D**) AANAT immunohistochemistry (IHC) at 1.5 dpc, 2.5 dpc, 3.5 dpc, and 4.5 dpc. (**E**) MT2 immunohistochemistry at 1.5 dpc, 2.5 dpc, 3.5 dpc, and 4.5 dpc. (**F**,**G**) Fertility assessment in the control (n = 10) and *Aanat* KO mice (n = 8); control (n = 9) and *Mt2* KO mice (n = 13); ** *p* < 0.01.

Quantitative real-time PCR (qPCR) showed that *Aanat* is dynamically expressed in the uteri of wild-type (WT) mice during pregnancy (Figure 1A). Immunohistochemistry (IHC) staining showed that *AANAT* was strongly expressed in the luminal epithelium, the glandular epithelium, and the stroma of the endometrium at 2.5 days post-coitus (dpc) while *AANAT* expression faded away at 4.5 dpc in the stroma but still presented in the luminal epithelium and the glandular epithelium (Figure 1D). *Mt1* expression was barely detectable in the uteri during pregnancy (Figure 1B). In contrast, *Mt2* was strongly expressed in the uteri during pregnancy (Figure 1C). Immunohistochemistry staining showed that *MT2* was strongly expressed in the luminal and glandular epithelia but had a weak stromal expression during early pregnancy (Figure 1E). 

To further explore the potential molecular mechanisms of uterus-generated melatonin on the physiology of endometrium during pregnancy, the *Aanat* and the *Mt2* KO mice were reconstructed via the CRISPR/Cas9 system. The F3 generation homozygotes of the KO mice were used in this study. The results showed that the average litter size (*Aanat* KO, 10.29 ± 0.81 vs. WT, 13.4 ± 0.6, Figure 1F; *Mt2* KO, 5.462 ± 0.56 vs. WT, 7.7 ± 0.441, Figure 1G) was significantly reduced in the KO mice compared with the WT ones (*p* < 0.05).

### 2.2. Effects of Aanat KO on Embryo Implantation and Uterine E2 and P4 as Well as Their Related Downstream Gene Responses at 4.5 dpc

**Figure 2:**
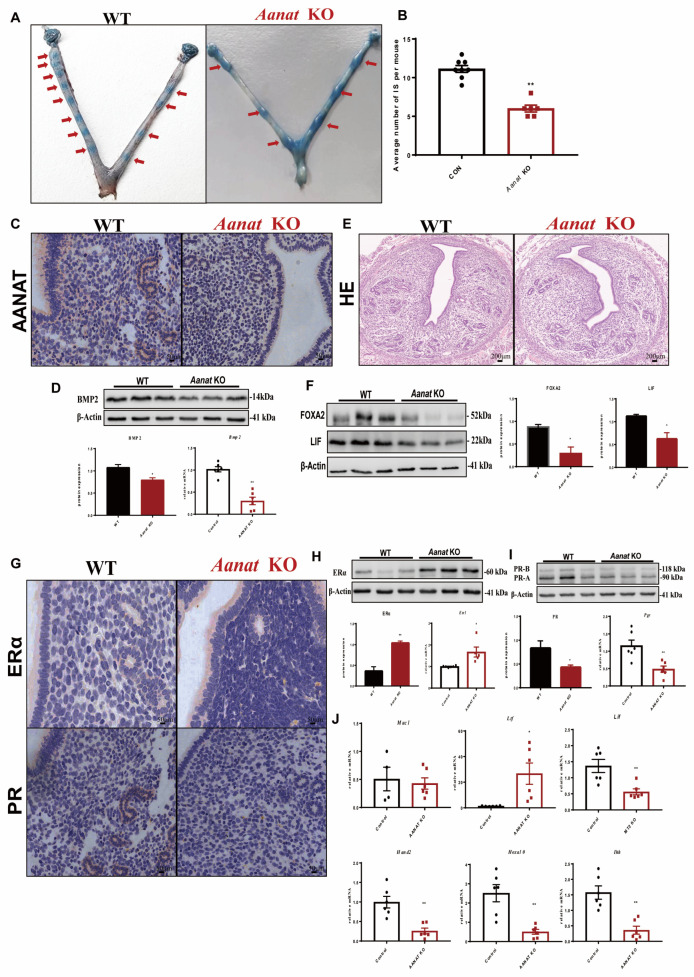
Effects of *Aanat* KO on endometrial receptivity and embryo implantation at 4.5 dpc in mice. (**A**,**B**) Implantation sites at 4.5 dpc, implantation window. As the red arrows indicate, the blue is the attachment sites displayed after Chicago Sky Blue staining. (**C**) Immunohistochemical staining of a pregnant uterus at 4.5 dpc; the brown staining indicates AANAT, and the blue staining indicates the nucleus. (**D**) Western blotting of BMP2 with statistical analysis. (**E**) HE staining of 4.5 dpc uteri. (**F**). Western blotting of the FOXA2 LIF protein with statistical analysis. (**G**) Immunohistochemical staining of a 4.5 dpc uterus; the brown signal is ERα and PR, and the blue signal is the nucleus. (**H**) Western blotting of the ERα protein with statistical analysis. (**I**) Western blotting of the PR-A protein with statistical analysis. (**J**) Expression of the downstream response genes of estrogen and progesterone by PCR, n = 6 Note: * *p* < 0.05, ** *p* < 0.01.

In order to determine the exact time period of embryo loss, the uteri were taken from pregnant mice at different timepoints. The results showed that on day 4.5 of pregnancy, i.e., after the adhesion reaction occurred during embryo implantation in early pregnancy, the number of implantation points in the *AANAT* knockout mice was significantly lower than that in the WT mice (Figure 2B), and only a small number of embryos could undergo implantation reactions compared to the WT mice (Figure 2A). 

Western blot analysis of FOXA2 indicated that the gland integrity in the uteri was disrupted in the *Aanat* KO mice compared to the WT mice (Figure 2F). However, the numbers of glands were not significantly different in the uteri between the KO and WT mice as measured by HE staining (Figure 2E).

The window of implantation in mice occurs at day 4 of pregnancy and is characterized by the transition from an E2-dominant proliferative state to a P4-responsive state [1]. Western blot and qPCR analyses showed an abnormal expression of implantation marker bone morphogenetic protein 2 (BMP2) on day 4.5 of pregnancy in the uteri of the KO mice (Figure 2D). Immunostaining, Western blot, and qPCR analyses of the day 4.5 of pregnancy uteri revealed a statistically significant upregulation of estrogen receptor α (ERα) and a significant downregulation of the progesterone receptor (PR) in the *Aanat* KO mice compared to the WT mice (Figure 2H,I). 

Then, the expressions of uterine receptivity marker genes including those responding to estrogen E2 (mucin 1 (*Muc1*) and lactoferrin (*Ltf*), leukemia inhibitory factor (*Lif*)), progesterone P4 (heart and neural crest derivatives expressed 2 (*Hand2*), homeobox A10 (*Hoxa10*), and Indian hedgehog homolog (*Ihh*)) were all analyzed. The expression of *Muc1* was similar between the WT and *Aanat* KO mice (Figure 2J). *Ltf* expression was significantly upregulated while *Lif* was downregulated in the uteri of the *Aanat* KO mice compared to the WT mice (Figure 2J). Moreover, the expression of all P4-target downstream genes including *Ihh* in the epithelium and *Hoxa10* and *Hand2* in the stroma was significantly downregulated in the *Aanat* KO mice compared to the WT mice (Figure 2J).

### 2.3. Effects of Aanat KO on Global Gene Expression in Uteri during the Window of Implantation at 4.5 dpc in Mice

**Figure 3:**
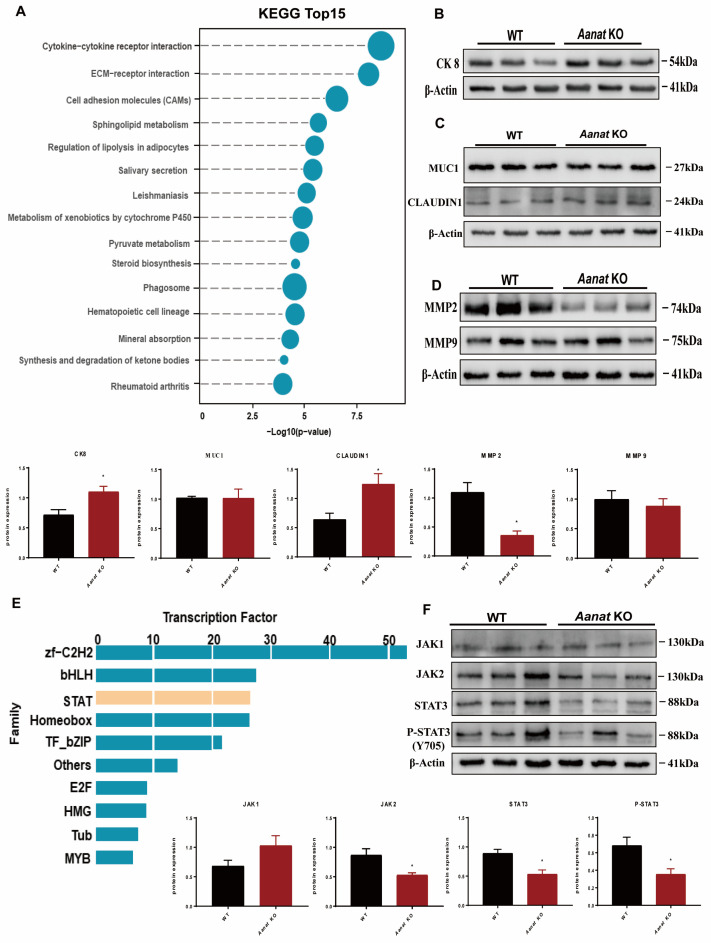
Effects of *Aanat KO* on the essential pathways of uterine implantation in the implantation window at 4.5 dpc in mice. (**A**) KEGG cluster analysis. (**B**–**D**) Western blotting of ECM, CK8, MMP2, MMP9, MUC1, and CLDN1, respectively, with statistical analyses. (**E**) Transcription factor differential analysis. (**F**) Western blotting of JAK–STAT with statistical analysis, n = 3. Note: * *p* < 0.05.

Transcriptome profiling of the implantation sites in the uteri at day 4.5 of pregnancy was conducted; the results showed that the expression of 1364 genes was differentiated (804 increased, 560 decreased) between the WT and *Aanat* KO mice. Among the 560 downregulated genes, the expression of uterus-related genes including *Ccl8*, *Il9*, *Il6st*, *Ccl25*, *Il1f8*, *Eda2r*, *Csf2rb*, *Csf2rb2*, *Tnfrsf18*, *Itga2*, *Thbs3*, *Col6a6*, *Sv2c*, *Sv2b*, and *Tnr* was significantly lower in the *Aanat* KO mice than in the WT mice (Supplementary Table S1). Functional analysis found that the differentially expressed genes were enriched in the cytokine−cytokine receptor interaction and the ECM−receptor interaction (Figure 3A). 

Functional analysis showed that 804 upregulated genes in the *Aanat* KO mice were enriched for the pathways associated with infectious diseases, cancers, neurodegenerative diseases, endocrine and metabolic diseases, immune system, signal transduction, and translation. Many of those genes are known to be related to enzymes (*Ass1*, *Fads3*), forkhead box transcription factors (*Foxo1*, *Foxo3*, *Foxo6*, *Foxr1*, *Foxr2*, *Foxp4*, *Foxn4*, *Foxm1*, *Foxl1*, *Foxl2*, *Foxi3*, *Foxf2*, *Foxc1*, *Foxc2*), and decidualized stromal cells, such as members of the prolactin (PRL) family (*Prl8a2*, *Prl6a1*) (Supplementary Table S2).

### 2.4. Effects of Aanat KO on the ECM−Receptor Interaction at 4.5 dpc in Mice

The protein expression level of CK8 was significant increased in uteri of both the WT and *Aanat* KO mice on day 4.5 of pregnancy. We evaluated mucin 1 (MUC1) and claudin 1 (CLDN1) as the components of epithelial adherence junctions and tight junctions, respectively. The expression level of MUC1 was similar between the WT and *Aanat* KO mice, but the expression of CLDN was significantly increased in the *Aanat* KO mice compared to the WT mice (Figure 3C).

Basement membrane remodeling enzymes Mmp-2 and Mmp-9 in stromal cells at the implantation sites were also influenced by KO, i.e., *Aanat* KO significantly downregulated the expression of Mmp-2 but kept the expression of Mmp-9 similar to that of the WT mice (Figure 3D).

### 2.5. Effects of Aanat KO on STAT Signaling at 4.5 dpc in Mice

Functional analyses of the 1364 differential genes of the day 4.5 of pregnancy uteri identified 257 differential transcription factors (139 up, 118 down). Members of the STAT family including *Ctsl*, *Eif4ebp1*, *Prr5l*, *Xpnpep2*, *Stx19*, *Prcp*, *Jakmip1*, *Hnf1aos1* as well as *Stat3*, *Stat5b*, and *Stat6* were significantly downregulated in the *Aanat* KO mice compared to the WT mice (Figure 3E). The expression of forkhead family member *Foxa2*, known to be expressed uniquely in uterine glands, was also statistically significantly downregulated in the *Aanat* KO mice compared to the WT mice (Supplementary Table S3). JAK2, STAT3, and tyrosine (Y) 705-phosphorylated STAT3 (pSTAT3^Y705^) responsible for decidualization and epithelial remodeling in mice were also significantly downregulated (Figure 3F). 

### 2.6. Effects of Mt2 KO on Uterine E2 and P4 Responsiveness and Glands Formation at 4.5 dpc in Mice

**Figure 4:**
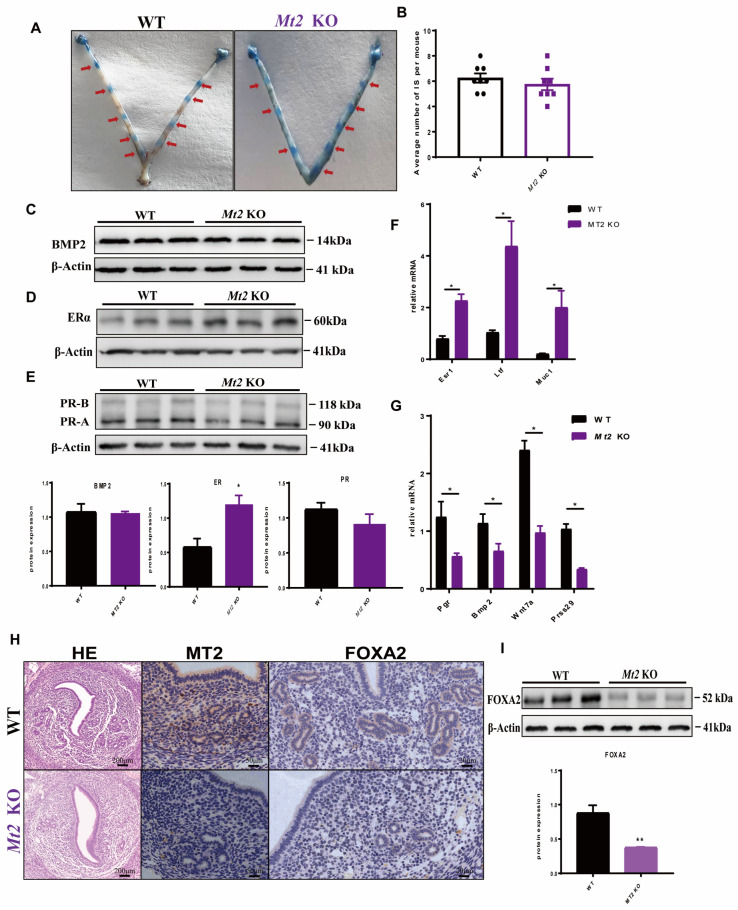
Effects of Mt2 KO on the uterine estrogen and progesterone response and uterine glands formation during the implantation window. (**A**,**B**) Implantation sites during the implantation window with statistical analysis. As the red arrows indicate, the blue is the attachment sites displayed after Chicago Sky Blue staining. (**C**) Expression of the BMP2 protein. (**D**) Expression of the ERα protein with statistical analysis. (**E**) Expression of the PR-A protein with statistical analysis. (**F**,**G**) Quantitative PCR analysis of estrogen and progesterone response-related genes. (**H**) HE staining of the uteri at 4.5 day of pregnancy; immunohistochemical staining of the uterus in 4.5 dpc mice; the brown staining is MT2 and the blue staining is the nucleus. (**I**) Expression of FOXA2 with statistical analysis, n = 3. Note: * *p* < 0.05, ** *p* < 0.01.

Based on the expression patterns of *Mt1/2* in the uterus mentioned above, the *Mt2* KO mice were selected to test their responses to early pregnancy. The results showed that at 4.5 dpc, the number of implantation sites in the knockout mice was not significantly lower than in the WT mice (Figure 4A,B). Therefore, the decrease in the litter size of *Mt2* KO mice may not occur at day 4.5 of gestation but may occur after the implantation window.

When the response of uterine estrogen and progesterone in the window of implantation was examined, it was found that the level of ER protein expression was significantly elevated (Figure 4D), while PR (Figure 4E) and BMP2 (Figure 4C) expression in the *Mt2* KO mice was comparable to those of the WT mice. The expression of uterine receptivity marker genes including those responding to estrogen E2 (estrogen receptor α (*Esr1*), *Ltf*, Muc1), progesterone P4 (progesterone receptor (Pgr), Bmp2, Wnt family member 7a (Wnt7a), and serine protease 29 (Prss29)) were significantly downregulated in the *Mt2* KO mice compared to the WT mice (Figure 4F,G). 

HE staining and immunostaining analyses indicated that the genesis of endometrial glands in the uterus is compromised by *Mt2* KO (Figure 4H). The expression of FOXA2 was significantly downregulated in the *Mt2* KO mice compared to the WT mice (Figure 4I). 

### 2.7. Effects of Mt2 KO on Abortion and Uterine Immunity during the Second Trimester of Pregnancy

**Figure 5:**
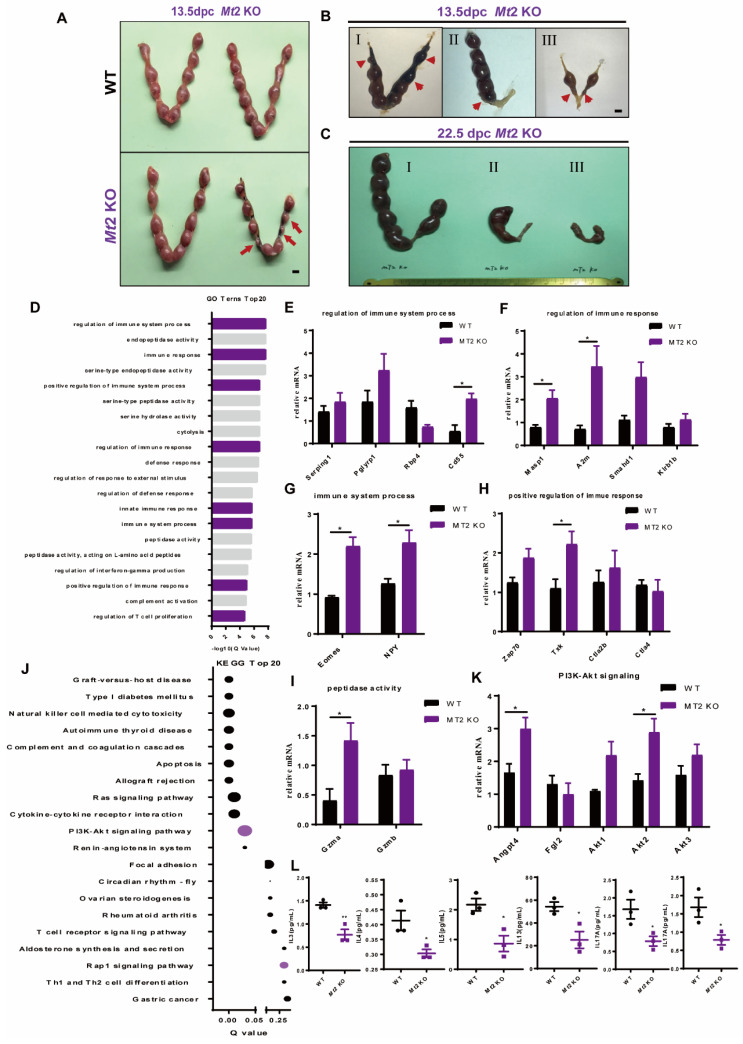
Effects of *Mt2* KO on embryo loss and abnormal uterine immune function at 13.5 dpc in mice. (**A**–**C**) Uterine morphology. The red arrows indicate atrophy of implantation sites. I–III shows different degrees of morphological abnormalities. (**D**) GO enrichment analysis. (**E**–**I**) QPCR validation of GO term-related genes in uteri of 13.5 dpc (n = 3). (**J**) KEGG enrichment analysis. (**K**) QPCR validation of KEGG term-related genes in uteri of 13.5 dpc (n = 3). (**L**) Cytokines in uteri of 13.5 dpc (n = 3). Note: * *p* < 0.05, ** *p* < 0.01.

Since there was no significant change in the number of attachment points in the *Mt2* KO mice during the window of implantation, the attachment points in the second trimester were examined. The results showed that some of the *Mt2* KO mice had hemorrhage in their implantation sites and even abortion on day 13.5 of pregnancy (Figure 5A,B). The uteri were dissected on day 22.5 of pregnancy before the delivery. It was observed that the implantation sites of the uterus in the *Mt2* KO mice had different degrees of atrophy and morphological abnormalities, with a lower number of fetuses (Figure 5C).

Transcriptome profiling of implantation sites of the uteri and the placentae on day 13.5 of pregnancy identified 2285 differentiated genes (1570 up, 715 down) between the WT and *Mt2* KO mice (Supplementary Table S4). Based on the standard of log_2_ fold change > 2 and log_2_ fold change < −2, *p* < 0.05, the screened upregulated genes were enriched by GO (Supplementary Table S5). The results showed that 8 of the top 20 GO items were related to the immune response including regulation of immune system process signaling, regulation of immune response signaling, immune system process signaling, positive regulation of immune response signaling, and peptidase activity signaling (Figure 5D). QPCR analysis of the day 13.5 of pregnancy uteri and placentae indicated that complement decay-accelerating factor 55 (*Cd55*) (Figure 5E), mannose-associated serine protease 1 (*Masp1*) (Figure 5F), alpha-2-macroglobulin (*A2m*) (Figure 5F), eomesodermin (*Eomes*) (Figure 5G), neuropeptide Y (*Npy*) (Figure 5G), tyrosine kinase (*Txk*) (Figure 5H), and granzyme A (*Gzma*) (Figure 5I) were significantly upregulated in the *Mt2* KO mice compared to the WT mice. 

The differentially expressed genes enriched by KEGG were also analyzed (Figure 5J), and the results showed that the differentially expressed genes were enriched in the PI3K–Akt pathway and immune-related pathways (Supplementary Table S6). The expression of angiopoietin 4 (*Angpt4*) and serine/threonine kinase 2 (*Akt2*) was significantly increased in the *MT2* KO mice compared to the WT mice (Figure 5K). In contrast, the expression of 21 cytokines decreased (Supplementary Figure S1); among them, the expression of IL3, IL4, IL5, IL13, IL17, and GMSCF significantly decreased in the *Mt2* KO mice compared to the WT mice (Figure 5L).

## 3. Discussion

It is well-documented that melatonergic systems including the rate-limiting synthetic enzyme of AANAT and its membrane receptors MT1 and MT2 in animal reproductive systems are essential for successful pregnancy [14,15,16,36,40,43]. However, there is a lack of evidence to confirm whether the uterine local melatonergic system is involved in endometrial receptivity and implantation during the implantation window, i.e., the early stage of pregnancy. First, we identified that *Aanat*, *Mt1*, and *Mt2* were expressed in the uteri of the mice. Compared to the strong expression of *Aanat* and *Mt2*, the expression of *Mt1* in the uteri of the mice was considerably weak; therefore, in the following studies, we generated *Aanat* and *Mt2* KO mice to systematically investigate the effects of the uterine melatonergic system on early pregnancy. The results showed that the implantation sites of fetuses were significantly reduced in the *Aanat* or *Mt2* KO mice compared to the WT mice, indicating the importance of the melatonergic system in successful early pregnancy.

The endometrium of the *Aanat* KO mice at 4.5 dpc showed a hyper-estrogenic status, with an increased expression of E2-regulated genes (*Lif*, *Ltf*, *Muc1*) and unopposed luminal epithelial cell proliferation. These abnormalities appeared to be associated with the downregulation of PR and other PR-regulated genes including *Hand2*, *Hoxa10*, and *Ihh.* The results indicated that there was an endometrial hypersensitivity to E2 and a defective PR function during the window of implantation, and these alterations jeopardized the ability of the endometrium to reach the receptive phase in the *Aanat* KO mice. 

At the molecular level, STAT signaling was altered in the endometrial tissues of the *Aanat* KO mice, including the downregulation of *JAK2*, *STAT3*, and tyrosine (Y) 705-phosphorylated STAT3 (*pSTAT3Y705*). In this signaling pathway, cytokine interactions can recruit several receptors, which causes Janus kinases, also known as JAKs, to cluster together [48]. These JAKs can perform cross-phosphorylation and activation of one another as well as of particular cytoplasmic domains on each of their individual cytokine receptors. Thus, the STAT3 pathway is positively involved in the invasion, proliferation, and/or differentiation of trophoblast cells and facilitates pregnancy-related activities including implantation [48,49]. The activation of STAT3 usually occurs during the early post-implantation stage and is necessary for embryonic development [50]. This situation can be corrected by replacing with STAT3b, an alternative splice form of STAT3 [51]. Takeda et al. reported that STAT3 is expressed on the extraembryonic visceral endoderm in mouse embryos at 7.5 dpc [52]. 

Furthermore, other important elements that are inhibited for successful early pregnancy are matrix metalloproteinases (MMPs). MMPs are a class of zinc (II)-dependent endopeptidases that can cleave extracellular matrix (ECM) proteins and facilitate angiogenesis and tissue remodeling [53]. The elevated expression of MMP-2 and MMP-9 is well-documented in the phases of healthy pregnancy [54]. A change in MMP-2 expression or activity may relate to EGF-mediated trophoblast invasion [55]. High levels of MMP-9 promote extravillous trophoblast cell invasion by the promotion of the breakdown of the endometrial ECM and the loosening of intercellular connections [55] However, this ECM−receptor interaction is jeopardized in *Aanat* KO mice. The expression of MMP-2 and MMP-9 in the *Aanat* KO mice was downregulated compared to the WT mice. These alterations may result in inadequate trophoblast invasion of the maternal endometrium with reduced uteroplacental perfusion for embryonic growth, and these were observed in the current study.

In the development of pregnancy, uterine glands also play critical biological roles [56,57]. LIF is only expressed by the uterine glandular epithelium (GE) during the window of receptivity to respond to the nidatory surge of estrogen from the ovaries on day 4 of pregnancy in mice. The lack of uterine glands or LIF-null animals including mice and sheep exhibits the infertile phenotype indicating that gland-derived substances are necessary for the development and maintenance of pregnancy [57,58,59,60]. For the fetus, its organ development is governed by transcription factors including forkhead box (FOX) [61]. FOXA2 is expressly expressed in the glands of the uterus in both mice and humans [58]. Conditional deletion of *Foxa2* with the progesterone receptor (Pgr)–Cre mouse model, which only ablates genes in the endometrial epithelium, the stroma, and the inner circular myometrium of the uterus after birth, compromises the development of endometrial glands in the neonatal uterus [58]. The FOXA2-deficient mouse was infertile due to defects in embryo attachment and a lack of LIF expression on day 4 of pregnancy [62]. Uterine glands are also colonized by extravillous trophoblast cells known as “endoglandular trophoblasts”, which are similar to the invasion of spiral arteries [63]. In fact, a lack of glandular activity can cause early pregnancy failure in mammals [64]. Our results showed that FOXA2 was significantly downregulated in the Aanat KO mice and the Mt2 KO mice at 4.5 dpc. HE and immunostaining showed that the genesis of uterine endometrial glands was compromised by Mt2 KO. The glandular dysfunction caused by the melatonergic system deficiency has a direct association with the reduced implantation sites observed in the study.

In the early interaction of the uterus and the embryo, the uterine immunological milieu must continue to be in an anti-inflammatory state after the initial challenge of implantation [10], and this immunological balance was impaired in the *Mt2* KO mice compared with the WT mice. Usually, the interactions between the stromal and vascular parts of the placenta and the uterus are controlled by a layer of maternal immune cells, the decidua [65]. In addition to promoting placental development and function, the decidua has a highly specialized structure to prevent the placenta from being attacked as an alien organ [65]. The decidua also contains immune cells that fight off infections. On the other hand, preterm delivery, intrauterine growth restriction (IUGR), recurrent spontaneous abortion (RSA), and preeclampsia are all associated with imbalanced decidual activity, for example, elevated decidual leukocytes [65] and cytokine release [66], which cause an inflammatory cascade in the uterus [12]. Melatonin is an anti-inflammatory molecule that can reduce the uterine inflammatory reaction and keep the placenta, the uterus, and the embryo healthy. *Aanat* KO reduces the local melatonin circulation and level in the uterus and impairs the anti-inflammatory function of the decidua. Many of the functions of melatonin are mediated by its membrane receptors MT1/2, and *Mt2* KO inevitably influences the anti-inflammatory activity of melatonin in the uterus. All of this was observed in the current study.

## 4. Materials and Methods

### 4.1. Ethic Statement

The Chinese Association for Laboratory Animal Sciences’ policies and guidelines were followed when conducting all of the animal experiments, which were authorized by the institution’s ethics committee.

### 4.2. Chemicals and Agents

The chemicals used in this experiment were purchased from Sigma-Aldrich Co. (St Louis, MO, USA) unless otherwise stated.

### 4.3. Animals

ICR mice were selected to perform the *Aanat* knockout (*Aanat* KO) [15]. C57BL/6 J mice were selected to perform the Mtnr1b knockout (*Mt2* KO) [16]. The *Aanat* and the *Mt2* KO mice were reconstructed via the CRISPR/Cas9 system. The guide RNA (gRNA) was designed using the fragment of the second *Aanat* exon. Single-guide RNA direct Cas9 endonuclease cleavage of exon2 of the *Mt2* gene was performed, and a DSB (double-strand break) was created. The pups were genotyped by PCR, followed by sequence analysis (Supplementary Table S7). In this study, 10–15-week-old mice were used. The specific implementation time of this experiment was from 1 September 2021 to 30 December 2022.

The approved protocol number for the study was AW91103202-1-1. 

### 4.4. Tissue Acquisition

The females were mated with the same-genotype male mice. The day with vaginal plug appearance was marked as 1 dpc. The uteri were collected at 1.5 days of pregnancy (1.5 dpc), 2.5 dpc, 3.5 dpc, 4.5 dpc, and 13.5 dpc, respectively, and the fetuses were stripped at 13.5 dpc. The uteri were weighted and then washed three times with ice-cold PBS. For each mouse, a portion of the uterus was fixed with 4% paraformaldehyde for immunohistochemistry. The remaining portion was stored at −80 °C for Western blotting, qPCR, or transcriptome sequencing.

### 4.5. Immunohistochemistry

The uteri were immersed in paraffin for 24 h and preserved in 4% paraformaldehyde before being sliced into 5 mm sections. When the sliced samples underwent dewaxing and hydrating, they were treated with xylene and alcohol (1:1 by volume), ethanol gradient solutions (90, 80, 50%), and water for 5 min consecutively. The slices were boiled for two minutes, then submerged into a citric acid–disodium hydrogen phosphate buffer. The samples were treated with 30% hydrogen peroxide in the dark for 15 min and washed with PBS. The anti-AANAT antibody (1:200 dilution; ab3505; Abcam, Cambridge, UK), anti-estrogen receptor alpha antibody (1:200 dilution; ab32063; Abcam, Cambridge, UK), anti-progesterone receptor A/B antibody (1:500 dilution; 8757; CST, Boston, MA, USA), anti-FOXA2 (1:500 dilution; ab108422; Abcam, Cambridge, UK), and anti-MT2 antibody (1:100 dilution; ab203346; Abcam, Cambridge, UK) were incubated with these slices overnight at 4 °C. After a wash with PBS, the samples were incubated with biotinylated goat anti-rabbit IgG (1:200 dilution; ZB2050; Zsbio, Beijing, China) for 1 h at room temperature. Following the addition of the ABC complex (Vector Laboratories, Burlingame, CA, USA) for 2 h at room temperature, the presence of peroxidase activity was determined by staining with diaminobenzidine (Sigma, St Louis, MO, USA) for less than 30 s. The negative control involved the use of PBS to replace the first antibody. Following dehydration and counterstaining with hematoxylin, the sections were coated with coverslips. An optical microscope was used to capture the images of the uteri with immunohistochemistry (Olympus microscope CX41, Olympus, Tokyo, Japan). The working distance was around 0.56 mm, the objective lens’s magnification was 40×, and the numerical aperture was 0.65.

### 4.6. Western Blot

The uteri (120 mg) were sliced into small pieces and sonicated (40% AMPL, 30 s pulse on, 30 s pulse off, 3 min) in an immunoprecipitation assay (RIPA, 100 mg/mL) buffer containing 1 mmol/L phenylmethyl sulfonyl fluoride (PMSF). The samples were stained with Coomassie blue. The proteins isolated from the samples were boiled in a loading buffer for 10 min and the protein lysates (approximately 50 μg) were electrophoresed on 12% SDS-PAGE and then transferred onto polyvinylidene difluoride (PVDF) membranes (Bio-Rad Laboratories, Richmond, CA, USA; U = 20 V, 0.1 mA). The anti-AANAT antibody (1:2000 dilution; ab3505; Abcam, Cambridge, UK), anti-MT2 antibody (1:500; ab203346; Abcam), anti-ERα antibody (1:500; ab32063; Abcam, Cambridge, UK), anti-PR antibody (1:500; 8757; CST, Boston, MA, USA), anti-BMP2 antibody (1:1000; ab214821; Abcam, Cambridge, UK), anti-FOXA2 antibody (1:1000; ab108422; Abcam, Cambridge, UK), anti-cytokeratin 8 antibody (1:20,000; ab53280; Abcam, Cambridge, UK), anti-MMP2 antibody (1:2000; ab86607; Abcam, Cambridge, UK), anti-MMP9 antibody (1:1000; ab283575; Abcam, Cambridge, UK), anti-MUC1 antibody (1:2000; ab109185; Abcam, Cambridge, UK), anti-claudin 1 antibody (1:1000; 13050-1-AP; Proteintech, Wuhan, China), anti-JAK1 antibody (1:1000; ab133666; Abcam, Cambridge, UK), anti-JAK2 antibody (1:1000; ab108596; Abcam, Cambridge, UK), anti-STAT3 antibody (1:1000; ab68153; Abcam, Cambridge, UK), anti-STAT3 (phospho Y705) antibody (1:2000; ab76315; Abcam, Cambridge, UK), anti-LIF antibody (1:500; 26757-1-AP; Proteintech, Wuhan, China), and anti-β-actin antibody (1:5000; AF5003; Beyotime, Shanghai, China) were incubated with the samples at 4 °C overnight. The membranes were then washed three times in Tris-buffered saline with Tween 20 (TBST) and incubated with horseradish peroxidase (HRP)-conjugated anti-rabbit antibody. Analysis was performed with enhanced chemiluminescence detection reagents (Applygen Technologies, Inc., Beijing, China) according to the manufacturers’ instructions. Protein bands were analyzed with ImageJ software (version 1.45; National Institutes of Health).

### 4.7. RNA Extraction and Real-Time qPCR

Total RNA was extracted from mouse ovaries using the TRIzol reagent (TaKaRa, Dalian, China) following the manufacturer’s instructions. The amount of RNA was determined by calculating the ratio of absorbance at 260 nm to the absorbance at 280 nm. Using the Prime Script RT reagent kit (TaKaRa Bio, Inc., Tokyo, Japan), cDNA was produced after reverse transcription of RNA. The amplification was performed using Light Cycler 480 SYBR.

For real-time qPCR, the NCBI website (https://www.ncbi.nlm.nih.gov/) included the gene sequence, and Primer Premier (version 5.00) was used to create the primers. SYBR Green (10 L), forward and reverse primers (0.75 M), template cDNA (2 L, 500–1000 ng), and ddH_2_O were the main components of the real-time qPCR processes with 40 cycles of 95 °C for 10 s and 60–62 °C for 1 min, followed by 10 min of 95 °C. ABI PRISM 7500 Sequence Detection System (Applied Biosystems, Foster, CA, USA) software was used to calculate the relative abundance of genes, and the 2^−ΔΔct^ technique was employed to calculate the gene expression. Actin-beta (Actb), a common housekeeping protein, served as the standard for normalization. Supplementary Table S7 contains a list of primer sequences. The experiments were replicated at least three times.

### 4.8. RNA-Seq Analysis

The uteri of the *Aanat* KO mice and the *Mt2* KO mice were collected at 4.5 dpc or 13.5 dpc. The samples were analyzed by Annoroad Gene Technology. The total amount of 2 mg RNA per sample was used as the input material for the RNA sample preparations. Sequencing libraries were generated using NEBNext Ultra RNA Library Prep Kit for Illumina (#E7530L, NEB, Ipswich, MA, USA) following the manufacturer’s recommendations, and index codes were added to attribute sequences to each sample. The libraries were sequenced using NovaSeq 6000 (paired-end, 150 bp). All the reads were mapped to the reference genome of *Mesocricetus auratus* using HISAT2 v.2.2.1 [67]. RNA counts were generated from Feature Counts (v.2.0.0) [67]. Differential expression analysis was conducted using the edgeR package (v.3.34.1) [68].

## 5. Conclusions 

**Figure 6:**
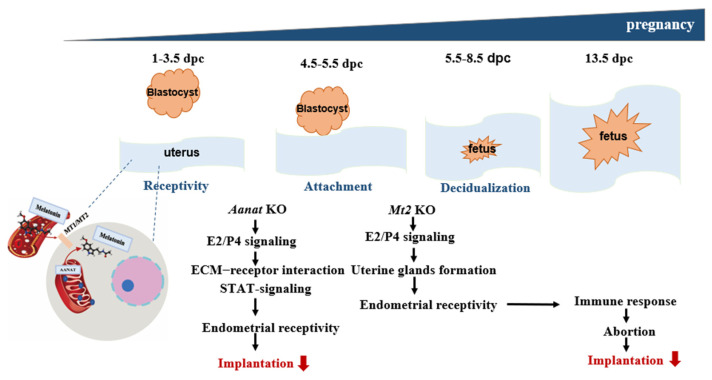
Schematic of the melatonergic systems (AANAT, MT2) that are active during embryo implantation.

The main results of our study are graphically presented in Figure 6. To the best of our knowledge, this is the first study to highlight the importance of an intact uterine melatonergic system for successful early implantation. Moreover, our results show significant placental maldevelopment and reduced early implantation sites in AANAT and MT2 KO mice. The melatonergic system potentially mediates the normal endometrial response to the E2 surge and P4. These mechanisms are essential for endometrial remodeling, which is essential to support embryo implantation, maintain early pregnancy, and balance the uterine immune response. Comprehensive exploration and determination of the biological role of the intrauterine AANAT-mediated melatonergic system in enhancing nidatory E2 surge- and P4-dependent endometrial receptivity also seems to be indispensable for eliminating the impairments in peri-implantation molecular communication between the endometrium, the placenta, and conceptuses following the transfer of mammalian embryos generated by such assisted reproductive technologies as standard in vitro fertilization [69,70], intracytoplasmic sperm injection [71,72], and somatic cell nuclear transfer [73,74]. Further studies on the local uterine melatonergic system are required to support the findings of our investigation.

## Data Availability

Not applicable.

## Supplementary Materials

The supporting information can be downloaded at: https://www.mdpi.com/article/10.3390/ijms24087127/s1.

## Abbreviations

AANAT	aralkylamine N-acetyltransferase
MT1	melatonin receptor 1A
MT2	melatonin receptor 1B
E2	estrogen
P4	progesterone
Erα (*Esr1*)	estrogen receptor α
PR (*Pgr*)	progesterone receptor
PTL	Preterm labor
PE	preeclampsia
IUGR	intra-uterine growth restriction
RSA	recurrent spontaneous abortion
GPCR	G protein-coupled receptor
SOD	superoxide dismutase
CAT	catalase
HB-EGF	heparin-binding epidermal growth factor-like growth factor
IVFET	In Vitro Fertilization and Embryo Transfer
BMP2	bone morphogenetic protein 2
*Lif*	Leukemia Inhibitory Factor
*Muc1*	mucin 1
*Ltf*	lactoferrin
*Hand2*	heart-and neural crest derivatives-expressed 2
*Hoxa10*	Homeobox A10
*Ihh*	Hedgehog Homolog Indian
Prss29	serine protease 29
Wnt7a	Wnt family member 7a
*Cd55*	complement decay-accelerating factor 55
*Masp1*	mannose associated serine protease 1
*A2m*	alpha-2-macroglobulin
*Eomes*	eomesodermin
*Npy*	neuropeptide Y
*Txk*	tyrosine Kinase
*Gzma*	granzyme A
*Angpt4*	angiopoietin 4
*Akt2*	Serine/Threonine Kinase 2
MMPs	matrix metalloproteinases
ECM	extracellular matrix
GE	glandular epithelium
FOX	forkhead box

## References

[1] Wang H., Dey S.K. (2006). Roadmap to embryo implantation: Clues from mouse models. Nat. Rev. Genet..

[2] Norwitz E.R., Schust D.J., Fisher S.J. (2001). Implantation and the survival of early pregnancy. N. Engl. J. Med..

[3] Zinaman M.J., Clegg E.D., Brown C.C., O’Connor J., Selevan S.G. (1996). Estimates of human fertility and pregnancy loss. Fertil. Steril..

[4] Wilcox A.J., Weinberg C.R., O’Connor J.F., Baird D.D., Schlatterer J.P., Canfield R.E., Armstrong E.G., Nisula B.C. (1988). Incidence of early loss of pregnancy. N. Engl. J. Med..

[5] Monsivais D., Nagashima T., Prunskaite-Hyyrylainen R., Nozawa K., Shimada K., Tang S., Hamor C., Agno J.E., Chen F., Masand R.P. (2021). Endometrial receptivity and implantation require uterine bmp signaling through an acvr2a-smad1/smad5 axis. Nat. Commun..

[6] Finn C.A., Martin L. (1970). The role of the oestrogen secreted before oestrus in the preparation of the uterus for implantation in the mouse. J. Endocrinol..

[7] Wetendorf M., Demayo F.J. (2012). The progesterone receptor regulates implantation, decidualization, and glandular development via a complex paracrine signaling network. Mol. Cell. Endocrinol..

[8] Li Q., Kannan A., Demayo F.J., Lydon J.P., Cooke P.S., Yamagishi H., Srivastava D., Bagchi M.K., Bagchi I.C. (2011). The antiproliferative action of progesterone in uterine epithelium is mediated by hand2. Science.

[9] Dimitriadis E., White C.A., Jones R.L., Salamonsen L.A. (2005). Cytokines, chemokines and growth factors in endometrium related to implantation. Hum. Reprod. Update.

[10] Mor G., Cardenas I., Abrahams V., Guller S. (2011). Inflammation and pregnancy: The role of the immune system at the implantation site. Ann. N. Y. Acad. Sci..

[11] Robertson S.A., Chin P.Y., Glynn D.J., Thompson J.G. (2011). Peri-conceptual cytokines--setting the trajectory for embryo implantation, pregnancy and beyond. Am. J. Reprod. Immunol..

[12] Benner M., Feyaerts D., Garcia C.C., Inci N., Lopez S.C., Fasse E., Shadmanfar W., van der Heijden O., Gorris M., Joosten I. (2020). Clusters of tolerogenic b cells feature in the dynamic immunological landscape of the pregnant uterus. Cell Rep..

[13] Redman C.W., Sargent I.L. (2010). Immunology of pre-eclampsia. Am. J. Reprod. Immunol..

[14] He C., Wang J., Zhang Z., Yang M., Li Y., Tian X., Ma T., Tao J., Zhu K., Song Y. (2016). Mitochondria synthesize melatonin to ameliorate its function and improve mice oocyte’s quality under in vitro conditions. Int. J. Mol. Sci..

[15] Yang M., Tao J., Wu H., Guan S., Liu L., Zhang L., Deng S., He C., Ji P., Liu J. (2019). Aanat knockdown and melatonin supplementation in embryo development: Involvement of mitochondrial function and dna methylation. Antioxid. Redox Signal..

[16] Wang J., Zhu T., Ma X., Wang Y., Liu J., Li G., Liu Y., Ji P., Zhang Z., Zhang L. (2021). Melatonergic systems of aanat, melatonin, and its receptor mt2 in the corpus luteum are essential for reproductive success in mammalsdagger. Biol. Reprod..

[17] Slominski A., Baker J., Rosano T.G., Guisti L.W., Ermak G., Grande M., Gaudet S.J. (1996). Metabolism of serotonin to n-acetylserotonin, melatonin, and 5-methoxytryptamine in hamster skin culture. J. Biol. Chem..

[18] Slominski A., Pisarchik A., Semak I., Sweatman T., Wortsman J., Szczesniewski A., Slugocki G., Mcnulty J., Kauser S., Tobin D.J. (2002). Serotoninergic and melatoninergic systems are fully expressed in human skin. FASEB J..

[19] Finocchiaro L.M., Nahmod V.E., Launay J.M. (1991). Melatonin biosynthesis and metabolism in peripheral blood mononuclear leucocytes. Biochem. J..

[20] Bubenik G.A. (2002). Gastrointestinal melatonin: Localization, function, and clinical relevance. Dig. Dis. Sci..

[21] Itoh M.T., Ishizuka B., Kuribayashi Y., Amemiya A., Sumi Y. (1999). Melatonin, its precursors, and synthesizing enzyme activities in the human ovary. Mol. Hum. Reprod..

[22] Suofu Y., Li W., Jean-Alphonse F.G., Jia J., Khattar N.K., Li J., Baranov S.V., Leronni D., Mihalik A.C., He Y. (2017). Dual role of mitochondria in producing melatonin and driving gpcr signaling to block cytochrome c release. Proc. Natl. Acad. Sci. USA.

[23] Wang L., Feng C., Zheng X., Guo Y., Zhou F., Shan D., Liu X., Kong J. (2017). Plant mitochondria synthesize melatonin and enhance the tolerance of plants to drought stress. J. Pineal Res..

[24] Martin M., Macias M., Escames G., Leon J., Acuna-Castroviejo D. (2000). Melatonin but not vitamins c and e maintains glutathione homeostasis in t-butyl hydroperoxide-induced mitochondrial oxidative stress. FASEB J..

[25] Gobbi G., Comai S. (2019). Sleep well. Untangling the role of melatonin mt1 and mt2 receptors in sleep. J. Pineal Res..

[26] Reiter R.J., Tan D.X., Manchester L.C., Pilar T.M., Flores L.J., Koppisepi S. (2007). Medical implications of melatonin: Receptor-mediated and receptor-independent actions. Adv. Med. Sci..

[27] Sharan K., Lewis K., Furukawa T., Yadav V.K. (2017). Regulation of bone mass through pineal-derived melatonin-mt2 receptor pathway. J. Pineal Res..

[28] Barberino R.S., Menezes V.G., Ribeiro A., Palheta R.J., Jiang X., Smitz J., Matos M. (2017). Melatonin protects against cisplatin-induced ovarian damage in mice via the mt1 receptor and antioxidant activity. Biol. Reprod..

[29] Li Y., Zhang Z., He C., Zhu K., Xu Z., Ma T., Tao J., Liu G. (2015). Melatonin protects porcine oocyte in vitro maturation from heat stress. J. Pineal Res..

[30] Park H.J., Park J.Y., Kim J.W., Yang S.G., Jung J.M., Kim M.J., Kang M.J., Cho Y.H., Wee G., Yang H.Y. (2018). Melatonin improves the meiotic maturation of porcine oocytes by reducing endoplasmic reticulum stress during in vitro maturation. J. Pineal Res..

[31] He C., Ma T., Shi J., Zhang Z., Wang J., Zhu K., Li Y., Yang M., Song Y., Liu G. (2016). Melatonin and its receptor mt1 are involved in the downstream reaction to luteinizing hormone and participate in the regulation of luteinization indifferent species. J. Pineal Res..

[32] Soliman A., Lacasse A.A., Lanoix D., Sagrillo-Fagundes L., Boulard V., Vaillancourt C. (2015). Placental melatonin system is present throughout pregnancy and regulates villous trophoblast differentiation. J. Pineal Res..

[33] Jin J.X., Lee S., Taweechaipaisankul A., Kim G.A., Lee B.C. (2017). Melatonin regulates lipid metabolism in porcine oocytes. J. Pineal Res..

[34] Papis K., Poleszczuk O., Wenta-Muchalska E., Modlinski J.A. (2007). Melatonin effect on bovine embryo development in vitro in relation to oxygen concentration. J. Pineal Res..

[35] Sampaio R.V., Conceicao S., Miranda M.S., Sampaio L.F., Ohashi O.M. (2012). Mt3 melatonin binding site, mt1 and mt2 melatonin receptors are present in oocyte, but only mt1 is present in bovine blastocyst produced in vitro. Reprod. Biol. Endocrinol..

[36] Tian X., Wang F., Zhang L., He C., Ji P., Wang J., Zhang Z., Lv D., Abulizi W., Wang X. (2017). Beneficial effects of melatonin on the in vitro maturation of sheep oocytes and its relation to melatonin receptors. Int. J. Mol. Sci..

[37] Bahadori M.H., Ghasemian F., Ramezani M., Asgari Z. (2013). Melatonin effect during different maturation stages of oocyte and subsequentembryo development in mice. Iran J. Reprod. Med..

[38] Ishizuka B., Kuribayashi Y., Murai K., Amemiya A., Itoh M.T. (2000). The effect of melatonin on in vitro fertilization and embryo development in mice. J. Pineal Res..

[39] Tian X.Z., Wen Q., Shi J.M., Liang-Wang, Zeng S.M., Tian J.H., Zhou G.B., Zhu S.E., Liu G.S. (2010). Effects of melatonin on in vitro development of mouse two-cell embryos cultured in htf medium. Endocr. Res..

[40] Wang F., Tian X., Zhang L., Tan D., Reiter R.J., Liu G. (2013). Melatonin promotes the in vitro development of pronuclear embryos and increases the efficiency of blastocyst implantation in murine. J. Pineal Res..

[41] Nishihara T., Hashimoto S., Ito K., Nakaoka Y., Matsumoto K., Hosoi Y., Morimoto Y. (2014). Oral melatonin supplementation improves oocyte and embryo quality in women undergoing in vitro fertilization-embryo transfer. Gynecol. Endocrinol..

[42] He C., Wang J., Li Y., Zhu K., Xu Z., Song Y., Song Y., Liu G. (2015). Melatonin-related genes expressed in the mouse uterus during early gestation promote embryo implantation. J. Pineal Res..

[43] Ma W.G., Song H., Das S.K., Paria B.C., Dey S.K. (2003). Estrogen is a critical determinant that specifies the duration of the window of uterine receptivity for implantation. Proc. Natl. Acad. Sci. USA.

[44] Richter H.G., Hansell J.A., Raut S., Giussani D.A. (2009). Melatonin improves placental efficiency and birth weight and increases the placental expression of antioxidant enzymes in undernourished pregnancy. J. Pineal Res..

[45] Moghani-Ghoroghi F., Moshkdanian G., Sehat M., Nematollahi-Mahani S.N., Ragerdi-Kashani I., Pasbakhsh P. (2018). Melatonin pretreated blastocysts along with calcitonin administration improved implantation by upregulation of heparin binding-epidermal growth factor expression in murine endometrium. Cell J..

[46] Zhang L., Zhang Z., Wang F., Tian X., Ji P., Liu G. (2017). Effects of melatonin administration on embryo implantation and offspring growth in mice under different schedules of photoperiodic exposure. Reprod. Biol. Endocrinol..

[47] Okatani Y., Okamoto K., Hayashi K., Wakatsuki A., Tamura S., Sagara Y. (1998). Maternal-fetal transfer of melatonin in pregnant women near term. J. Pineal Res..

[48] Fitzgerald J.S., Poehlmann T.G., Schleussner E., Markert U.R. (2008). Trophoblast invasion: The role of intracellular cytokine signalling via signal transducer and activator of transcription 3 (stat3). Hum. Reprod. Update.

[49] Hilton D.J. (1999). Negative regulators of cytokine signal transduction. Cell. Mol. Life Sci..

[50] Duncan S.A., Zhong Z., Wen Z., Darnell J.J. (1997). Stat signaling is active during early mammalian development. Dev. Dyn..

[51] Akira S. (1999). Functional roles of stat family proteins: Lessons from knockout mice. Stem Cells.

[52] Takeda K., Noguchi K., Shi W., Tanaka T., Matsumoto M., Yoshida N., Kishimoto T., Akira S. (1997). Targeted disruption of the mouse stat3 gene leads to early embryonic lethality. Proc. Natl. Acad. Sci. USA.

[53] Duellman T., Warren C.L., Peissig P., Wynn M., Yang J. (2012). Matrix metalloproteinase-9 genotype as a potential genetic marker for abdominal aortic aneurysm. Circ. Cardiovasc. Genet..

[54] Dang Y., Li W., Tran V., Khalil R.A. (2013). Emmprin-mediated induction of uterine and vascular matrix metalloproteinases during pregnancy and in response to estrogen and progesterone. Biochem. Pharmacol..

[55] Jing M., Chen X., Qiu H., He W., Zhou Y., Li D., Wang D., Jiao Y., Liu A. (2022). Insights into the immunomodulatory regulation of matrix metalloproteinase at the maternal-fetal interface during early pregnancy and pregnancy-related diseases. Front. Immunol..

[56] Burton G.J., Jauniaux E., Charnock-Jones D.S. (2007). Human early placental development: Potential roles of the endometrial glands. Placenta.

[57] Spencer T.E. (2014). Biological roles of uterine glands in pregnancy. Semin. Reprod. Med..

[58] Jeong J.W., Kwak I., Lee K.Y., Kim T.H., Large M.J., Stewart C.L., Kaestner K.H., Lydon J.P., Demayo F.J. (2010). Foxa2 is essential for mouse endometrial gland development and fertility. Biol. Reprod..

[59] Kelleher A.M., Milano-Foster J., Behura S.K., Spencer T.E. (2018). Uterine glands coordinate on-time embryo implantation and impact endometrial decidualization for pregnancy success. Nat. Commun..

[60] Rosario G.X., Stewart C.L. (2016). The multifaceted actions of leukaemia inhibitory factor in mediating uterine receptivity and embryo implantation. Am. J. Reprod. Immunol..

[61] Kaestner K.H. (2010). The foxa factors in organogenesis and differentiation. Curr. Opin. Genet. Dev..

[62] Kelleher A.M., Peng W., Pru J.K., Pru C.A., Demayo F.J., Spencer T.E. (2017). Forkhead box a2 (foxa2) is essential for uterine function and fertility. Proc. Natl. Acad. Sci. USA.

[63] Huppertz B., Weiss G., Moser G. (2014). Trophoblast invasion and oxygenation of the placenta: Measurements versus presumptions. J. Reprod. Immunol..

[64] Salamonsen L.A., Edgell T., Rombauts L.J., Stephens A.N., Robertson D.M., Rainczuk A., Nie G., Hannan N.J. (2013). Proteomics of the human endometrium and uterine fluid: A pathway to biomarkerdiscovery. Fertil. Steril..

[65] Erlebacher A. (2013). Immunology of the maternal-fetal interface. Annu. Rev. Immunol..

[66] Shynlova O., Lee Y.H., Srikhajon K., Lye S.J. (2013). Physiologic uterine inflammation and labor onset: Integration of endocrine and mechanical signals. Reprod. Sci..

[67] Kim D., Paggi J.M., Park C., Bennett C., Salzberg S.L. (2019). Graph-based genome alignment and genotyping with hisat2 and hisat-genotype. Nat. Biotechnol..

[68] Liao Y., Smyth G.K., Shi W. (2014). Featurecounts: An efficient general purpose program for assigning sequence reads to genomic features. Bioinformatics.

[69] Won J., Lee D., Lee Y.G., Hong S.H., Kim J.H., Kang Y.J. (2023). The therapeutic effects and optimal timing of granulocyte colony stimulating factor intrauterine administration during ivf-et. Life Sci..

[70] Isa T., Somfai T., Oyadomari M., Fusho K., Touma S., Suzuki N., Kaneko H., Katagiri Y., Kikuchi K. (2022). Production of agu piglets after transfer of embryos produced in vitro. Anim. Sci. J..

[71] Torikai K., Shimizu K., Nagatomo H., Kasai M., Kato-Itoh M., Kamada Y., Shibasaki I., Jeon H., Kikuchi R., Wakayama S. (2023). Removal of sperm tail using trypsin and pre-activation of oocyte facilitates intracytoplasmic sperm injection in mice and rats. J. Reprod. Dev..

[72] Briski O., Salamone D.F. (2022). Past, present and future of icsi in livestock species. Anim. Reprod. Sci..

[73] Samiec M., Skrzyszowska M., Lipinski D. (2012). Pseudophysiological transcomplementary activation of reconstructed oocytes as a highly efficient method used for producing nuclear-transferred pig embryos originating from transgenic foetal fibroblast cells. Pol. J. Vet. Sci..

[74] Skrzyszowska M., Smorag Z., Slomski R., Katska-Ksiazkiewicz L., Kalak R., Michalak E., Wielgus K., Lehmann J., Lipinski D., Szalata M. (2006). Generation of transgenic rabbits by the novel technique of chimeric somatic cell cloning. Biol. Reprod..

