# Supplementary figures and images for: The Uterine Melatonergic Systems of AANAT and Melatonin Membrane Receptor 2 (MT2) Are Essential for Endometrial Receptivity and Early Implantation in Mice
Source: Int J Mol Sci. 2023 Apr 12;24(8):7127. doi: 10.3390/ijms24087127 (PMC10139237; doi:10.3390/ijms24087127)

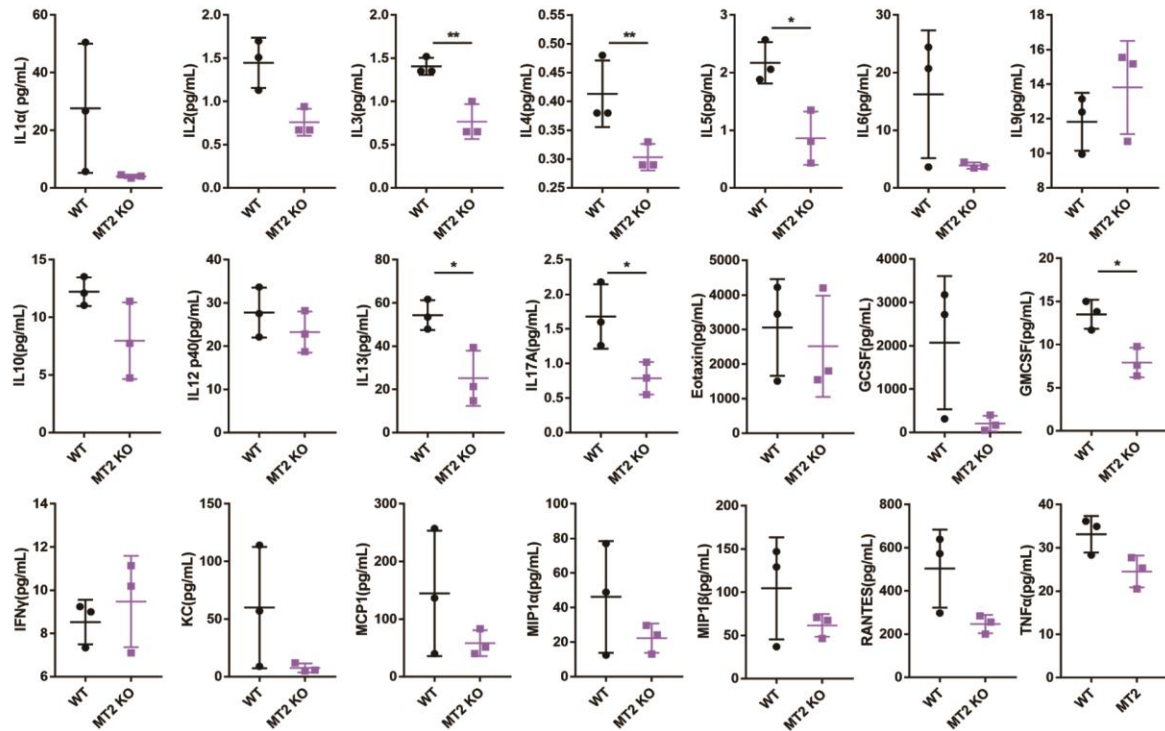

Supplementary Figure S1  
Cytokines in uteri of 13.5 dpc

Supplement: Supplementary file 1 [file ijms-24-07127-s001.zip › Figure S1.pdf]
